# Necrotizing Enterocolitis in a Term Newborn With Hypoxic-Ischemic Encephalopathy

**DOI:** 10.7759/cureus.106673

**Published:** 2026-04-08

**Authors:** Abdessamad Lalaoui, Ghizlane Kassal, Khalid Abi El Aala, Fatiha Bennaoui, Nadia El Idrissi Slitine, Fadl Mrabih Rabou Maoulainine

**Affiliations:** 1 Neonatal Intensive Care Unit, Mother and Child Hospital, Mohammed VI University Hospital Center, Marrakech, MAR; 2 Research Center for Childhood, Health and Sustainable Development, Faculty of Medicine and Pharmacy of Marrakesh (FMPM), Cadi Ayyad University, Marrakech, MAR

**Keywords:** enteral feeding, full-term neonate, necrotizing enterocolitis (nec), perinatal asphyxia, therapeutic hypothermia

## Abstract

Necrotizing enterocolitis (NEC) predominantly affects preterm infants and remains uncommon in term neonates. When it occurs in full-term newborns, it usually reflects underlying vulnerability or cumulative perinatal risk factors.

We report the case of a term male newborn (40 + 1 weeks’ gestation) who developed NEC in the context of multiple risk factors, including perinatal asphyxia with hypoxic-ischemic encephalopathy managed by therapeutic hypothermia, suspected early-onset neonatal infection requiring empirical antibiotics, and formula feeding. On day 4 of life, the infant presented with signs of an acute abdomen. Imaging revealed pneumoperitoneum, and surgery confirmed NEC with colonic perforation. Histopathology demonstrated inflammatory and hemorrhagic necrosis, and Hirschsprung’s disease was excluded. A colostomy was performed, with restoration of intestinal continuity at six months and normal neurodevelopment at 18 months.

This case highlights that NEC in term neonates, although rare, may result from the convergence of multiple perinatal insults, including hypoxic-ischemic injury and therapeutic hypothermia, and underscores the need for careful evaluation of predisposing factors that compromise intestinal integrity.

## Introduction

Necrotizing enterocolitis (NEC) is a severe neonatal gastrointestinal emergency characterized by intestinal inflammation and necrosis that may progress to perforation, sepsis, and death [1-3]. It occurs predominantly in preterm infants, representing approximately 1-7% of neonatal intensive care unit admissions, with a pooled incidence of about 7% among very-low-birth-weight infants [1,4]. In contrast, NEC is uncommon in full-term neonates, who account for only a small minority of cases, which underscores the clinical relevance of this entity in term infants [5].

The pathogenesis of NEC is multifactorial and involves a complex interaction between impaired intestinal perfusion, mucosal barrier disruption, abnormal bacterial colonization, and exaggerated inflammatory responses [1-3]. Whereas prematurity and intestinal immaturity are major drivers in preterm infants, NEC in term neonates is more often associated with identifiable predisposing conditions such as perinatal asphyxia, hypoxic-ischemic encephalopathy (HIE), congenital heart disease, sepsis, formula feeding, exchange transfusion, and other conditions that compromise intestinal oxygen delivery or mucosal integrity [2,5].

Among these factors, HIE is particularly relevant because perinatal hypoxia-ischemia may lead to systemic and mesenteric hypoperfusion followed by reperfusion injury, thereby promoting intestinal inflammation and barrier damage [6]. Therapeutic hypothermia is the standard neuroprotective treatment for neonates with moderate-to-severe HIE [7]. However, when gastrointestinal symptoms occur in this setting, NEC should remain an important diagnostic consideration, particularly in the presence of multiple concurrent perinatal insults.

NEC in term neonates remains insufficiently characterized, although reported cases tend to present early and may be associated with severe complications, including colonic involvement and perforation [5]. We report a term newborn with HIE treated with therapeutic hypothermia who developed severe NEC, highlighting the likely contribution of cumulative perinatal risk factors and the importance of early clinical vigilance in this rare but life-threatening condition.

## Case presentation

A term male neonate, born at 40 weeks + 1 day of gestation by vaginal delivery after 18 hours of labor, was admitted to the neonatal intensive care unit (NICU) for moderate HIE (Sarnat stage II). He was born to a 23-year-old primigravida after an uneventful, well-monitored pregnancy, with no history of gestational diabetes, hypertension, or other significant maternal illness. At birth, the infant showed signs of perinatal asphyxia, including delayed crying, low Apgar scores (1/10 at one minute and 4/10 at five minutes), and the need for resuscitation for 10 minutes. Birth weight was 3200 g (50th percentile), and length was 50 cm (50th percentile).

At one hour of life, arterial blood gas analysis showed severe metabolic acidosis (pH 7.02; lactate 13 mmol/L). Clinically, the newborn had generalized hypotonia, absent sucking reflex, and seizures at four hours of life, characterized by pedaling and boxing movements. Cerebral function monitoring showed type II convulsive activity according to the Al-Naqeeb classification [8]. Whole-body therapeutic hypothermia was initiated within the first six hours of life according to the institutional protocol, targeting a core temperature of 33-34°C for 72 hours, followed by controlled rewarming. A loading dose of phenobarbital was administered for seizure control.

Because C-reactive protein was elevated at 12 hours of life (21 mg/L), empirical antibiotic therapy with cefotaxime and gentamicin was started for suspected early-onset neonatal infection, as amoxicillin was unavailable at the time. During therapeutic hypothermia, the infant received minimal enteral feeding with formula milk because breast milk was not available, followed by gradual advancement of feeds after completion of cooling. The infant remained hemodynamically stable and did not require invasive mechanical ventilation or inotropic support.

On day 4 of life, shortly after completion of therapeutic hypothermia and during feed progression, the infant developed bilious vomiting, progressive abdominal distension, and cessation of stool passage. On physical examination, the abdomen was markedly distended and tense, with a stretched, shiny abdominal wall, mild tenderness on palpation, and absent bowel sounds on auscultation. In the setting of recent hypoxic-ischemic insult, formula feeding, and inflammatory deterioration, these findings raised concern for an acute abdominal process. The main diagnostic considerations were NEC, spontaneous intestinal perforation, Hirschsprung-associated enterocolitis, intestinal obstruction, and sepsis-related ileus.

**Figure 1:**
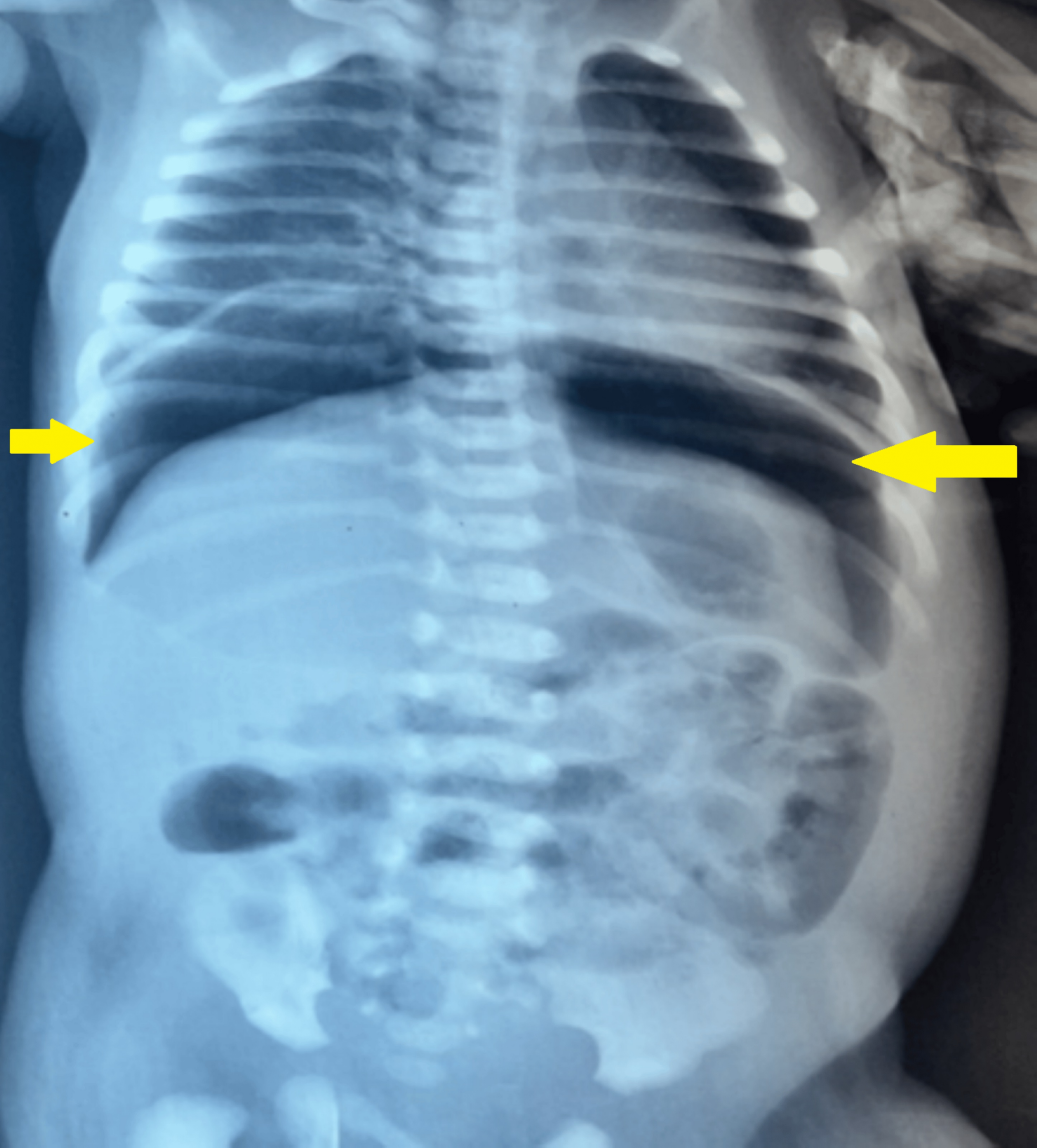
Abdominal X-ray on day 4 of life demonstrating a large pneumoperitoneum, with free intraperitoneal air outlining the abdominal cavity and bowel loops, consistent with intestinal perforation in the setting of advanced necrotizing enterocolitis Yellow arrows: Free intraperitoneal air beneath the diaphragms, consistent with pneumoperitoneum

Abdominal radiography demonstrated pneumoperitoneum (Figure 1).

Laboratory evaluation showed thrombocytopenia (104,000/mm³), hyponatremia (Na 133 mmol/L), metabolic acidosis (bicarbonate 11 mmol/L), and markedly elevated C-reactive protein (153 mg/L) (Table 1).

**Table 1:** Summary of laboratory findings related to the case GPT: Serum Glutamic Pyruvic Transaminase; GOT: Serum Glutamic Oxaloacetic Transaminase; CRP: C-reactive Protein

Variables	Patient Value at Admission	On day 4	Normal Range
pH	7.02	-	7.35-7.45
Base excess	-12	-	-4,+4
Lactate	13	2	<2
C-reactive protein (mg/L)	21	153	0.01–0.44
Leukocytes (/mm³)	14970	9,850	7,690–13,120
Hemoglobin (g/dL)	16	15.5	12.5–16.5
Platelets (/mm³)	132000	104,000	140,000–238,000
Prothrombin time (%)	97	98	70–100
Sodium (mEq/L)	135	133	131–143
Bicarbonates (mmol/L)	-	11	19–26
AST (GOT, U/L)	230	78	20–67
ALT (GPT, U/L)	159	30	9–25
Urea (g/L)	0.2	0.23	0.19–0.45
Creatinine (mg/dL)	3.92	4.2	0.33–0.93

These findings were highly suggestive of advanced NEC, compatible with Bell stage IIIB [9]. Echocardiography revealed no structural or hemodynamically significant congenital heart disease. Enteral feeding was discontinued, total parenteral nutrition was initiated, and broad-spectrum antibiotic therapy with imipenem, amikacin, and vancomycin was started.

**Figure 2:**
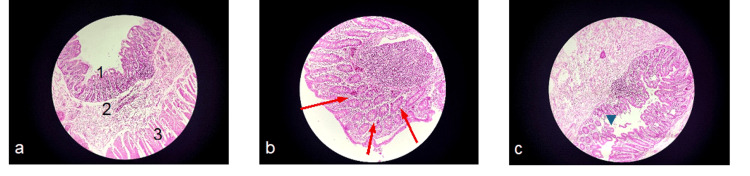
Histopathological findings of colonic tissue in necrotizing enterocolitis in our patient (a) Colorectal mucosa showing inflammatory changes without evidence of ganglion cell absence or nerve hypertrophy, excluding Hirschsprung disease. 1: Colorectal mucosa, 2: Submucosa, and 3: Muscularis propria (b) Colorectal mucosa with marked inflammatory remodeling. Red arrows indicate a polymorphous inflammatory infiltrate. (c) Colonic mucosa demonstrating ulcerative and inflammatory changes. The green arrow indicates mucosal ulceration.

Urgent exploratory laparotomy revealed perforation at the junction of the sigmoid and descending colon with purulent peritoneal fluid. Peritoneal fluid culture isolated Klebsiella pneumoniae resistant to third-generation cephalosporins and susceptible to imipenem and amikacin, as well as Enterococcus faecium susceptible to vancomycin; blood cultures remained sterile. Multiple segmental biopsies were obtained intraoperatively. Histopathological examination confirmed NEC, showing acute ulcerative and inflammatory lesions with hemorrhage and transmural necrosis of the intestinal wall. Normal colonic innervation was identified, thereby excluding Hirschsprung’s disease (Figure 2).

A diverting colostomy was performed. The postoperative course was favorable. Enteral feeding was restarted on postoperative day 5 and gradually advanced according to clinical tolerance. Intestinal continuity was restored at six months of age. At 18 months of follow-up, growth and psychomotor development were normal. Brain magnetic resonance imaging performed at one month of life was also normal.

## Discussion

NEC is predominantly a disease of prematurity, and its occurrence in a term neonate remains uncommon [1-3,5,10]. Unlike preterm NEC, which is largely driven by intestinal immaturity, NEC in full-term infants is more often associated with identifiable perinatal or neonatal stressors that impair intestinal perfusion, mucosal integrity, or host defense [1-3,5,10]. Reported risk factors include perinatal asphyxia, HIE, congenital heart disease, sepsis, formula feeding, exchange transfusion, and other conditions associated with intestinal hypoperfusion [1-3,5,10]. In the present case, NEC developed in the setting of several converging risk factors rather than as an isolated event.

The clinical course supports a multifactorial mechanism of intestinal injury. The infant experienced significant perinatal asphyxia, as reflected by low Apgar scores, prolonged resuscitation, severe metabolic acidosis, seizures, and moderate HIE. Hypoxic-ischemic injury is particularly relevant because it may induce systemic and mesenteric hypoperfusion followed by reperfusion-related mucosal damage, inflammatory activation, and impaired intestinal barrier function [1-3,6,10]. This sequence provides a plausible pathophysiological basis for NEC in term infants after perinatal distress. In the present case, intestinal vulnerability was likely further amplified by inflammatory stress, empirical antibiotic exposure, and formula feeding in the absence of human milk.

Therapeutic hypothermia should be interpreted cautiously in this context. It remains the standard neuroprotective treatment for eligible neonates with moderate-to-severe HIE [7]. However, gastrointestinal deterioration occurring during or shortly after cooling may create diagnostic uncertainty. In this case, therapeutic hypothermia should not be viewed as a direct causal factor in isolation, but rather as part of a broader clinical context in which multiple concurrent insults may have contributed to intestinal injury. This distinction is important because the observed temporal association does not establish causality.

The presentation of our patient is also consistent with previously reported features of NEC in term infants. Term NEC has been described as presenting early, often within the first week of life, and may be associated with more prominent colonic involvement than the classic ileal-predominant form seen in premature infants [5,10]. In our patient, NEC became clinically evident on day 4 of life and involved perforation at the sigmoid-descending colon junction, which is compatible with this reported phenotype. The disease was already advanced at diagnosis, with pneumoperitoneum and perforation present at the time of imaging, which limited the ability to characterize earlier, subtler prodromal signs. Nevertheless, the progression from feed exposure and abdominal distension to bilious vomiting, absent bowel sounds, inflammatory worsening, and radiographic perforation represents a clinically recognizable sequence that may help identify similar cases.

An important aspect of this case is the systematic exclusion of alternative diagnoses. Because colonic NEC in a term infant may mimic or coexist with Hirschsprung-associated enterocolitis, histopathological demonstration of normal colonic innervation was particularly relevant [11]. Likewise, the absence of structural or hemodynamically significant congenital heart disease helped narrow the etiological context and supports the interpretation of NEC as related primarily to cumulative perinatal injury rather than to a major cardiac low-flow state [5].

Compared with previously reported cases of term NEC, our patient shared several common features, including early onset, identifiable perinatal stress, severe presentation, and need for surgical management [5,10]. However, this case is notable for the coexistence of moderate HIE treated with therapeutic hypothermia, formula feeding due to lack of breast milk, positive peritoneal fluid cultures, colonic perforation with histological confirmation of NEC, and exclusion of Hirschsprung’s disease. The favorable long-term outcome is also noteworthy, with restoration of intestinal continuity at six months and normal neurodevelopment at 18 months despite the severity of the initial presentation.

This report should nevertheless be interpreted within its limitations. As this is a single case with multiple concurrent risk factors, no causal relationship can be established, particularly regarding the role of therapeutic hypothermia. In addition, because the diagnosis became clinically evident at an advanced stage, after perforation had already occurred, earlier subtle warning signs could not be fully characterized.

Despite these limitations, this case reinforces an important clinical message: in term neonates with HIE or other significant perinatal insults, gastrointestinal symptoms such as feeding intolerance, progressive abdominal distension, bilious vomiting, delayed stool passage, or reduced bowel sounds should not be attributed solely to nonspecific ileus or critical illness. NEC should remain an important diagnostic consideration, particularly when multiple risk factors coexist [1,5,10]. Early recognition, close monitoring, and timely escalation to imaging and surgical assessment are essential to improve outcomes in this uncommon but potentially life-threatening condition [9,10].

## Conclusions

NEC remains a rare but potentially life-threatening condition in term neonates. This case illustrates that severe NEC may occur in the setting of multiple concurrent perinatal and early postnatal risk factors, including HIE, formula feeding, inflammatory stress, and therapeutic hypothermia as part of the clinical context. Although no causal relationship can be established from a single case, this report highlights the importance of close monitoring and early evaluation of gastrointestinal symptoms in term neonates with significant perinatal insults to enable prompt diagnosis and timely intervention.
